# Balanced volatile sedation with isoflurane in critically ill patients with aneurysmal subarachnoid hemorrhage – a retrospective observational study

**DOI:** 10.3389/fneur.2023.1164860

**Published:** 2023-06-22

**Authors:** Martin B. Müller, Nicole A. Terpolilli, Susanne M. Schwarzmaier, Josef Briegel, Volker Huge

**Affiliations:** ^1^Department of Anaesthesiology, University Hospital, LMU Munich, Munich, Germany; ^2^Department of Neurosurgery, University Hospital, LMU Munich, Munich, Germany; ^3^Department of Critical Care and Anaesthesiology, Schön Klinik Bad Aibling, Bad Aibling, Germany

**Keywords:** balanced anesthesia, inhalation anesthetic, isoflurane, bispectral index (BIS), neurocritical care, subarachnoid hemorrhage

## Abstract

**Introduction:**

In patients with severe aneurysmal subarachnoid hemorrhage (SAH) deep sedation is often used early in the course of the disease in order to control brain edema formation and thus intracranial hypertension. However, some patients do not reach an adequate sedation depth despite high doses of common intravenous sedatives. Balanced sedation protocols incorporating low-dose volatile isoflurane administration might improve insufficient sedation depth in these patients.

**Methods:**

We retrospectively analyzed ICU patients with severe aneurysmal SAH who received isoflurane in addition to intravenous anesthetics in order to improve insufficient sedation depth. Routinely recorded data from neuromonitoring, laboratory and hemodynamic parameters were compared before and up to 6 days after initiation of isoflurane.

**Results:**

Sedation depth measured using the bispectral index improved in thirty-six SAH patients (−15.16; *p* = 0.005) who received additional isoflurane for a mean period of 9.73 ± 7.56 days. Initiation of isoflurane sedation caused a decline in mean arterial pressure (−4.67 mmHg; *p* = 0.014) and cerebral perfusion pressure (−4.21 mmHg; *p* = 0.013) which had to be balanced by increased doses of vasopressors. Patients required increased minute ventilation in order to adjust for the increase in PaCO_2_ (+2.90 mmHg; *p* < 0.001). We did not detect significant increases in mean intracranial pressure. However, isoflurane therapy had to be terminated prematurely in 25% of the patients after a median of 30 h due to episodes of intracranial hypertension or refractory hypercapnia.

**Discussion:**

A balanced sedation protocol including isoflurane is feasible for SAH patients experiencing inadequately shallow sedation. However, therapy should be restricted to patients without impaired lung function, hemodynamic instability and impending intracranial hypertension.

## 1. Introduction

Continuous management of pain, delirium and achievement of conscious sedation have evolved as a pillow of modern critical care therapy (1–3). However, patients with severe aneurysmal subarachnoid hemorrhage (SAH) often require initial deep sedation in order to curb brain edema formation, control cerebral oxygen consumption, and increase convulsive threshold in an attempt to improve cerebral outcome (4–6).

Recently volatile sedation has attracted increased attention in critical care medicine, as newly developed vaporizing systems have made their use safe and feasible outside the operating room (OR) (7, 8). Volatile sedation with its ability to lower the cerebral metabolic rate of oxygen (CMRO_2_) and shorter awakening and extubation times, offers some potentially desirable features in SAH patients in which prolonged deep sedation is often times needed (7–12). Notably, increasing evidence from experimental animal studies suggests that inhalational anesthetics such as isoflurane have a neuroprotective effect on ischemic brain tissue and positive and intrinsic effects on the occurrence and severity of cerebral vasospasm (13–18). In line with these data, recent studies identified the use of volatile anesthetics during aneurysm repair as an independent predictor of less angiographic vasospasm and less delayed cerebral ischemia in SAH patients (19, 20).

However, the clinical role of volatile sedation in SAH patients in the Neurosurgical Intensive Care Unit (NICU) remains limited and controversial, as a study from Purrucker et al. showed a critical increase in intracranial pressure (ICP), presumably caused by hemodynamic instability following initiation of monotherapy with sevoflurane in patients with acute brain injury (21). In accordance with these findings, short-term use of volatile anesthetics in the OR was associated with episodes of increased ICP either caused by a decrease in mean arterial pressure (MAP) and cerebral perfusion pressure (CPP) or increased regional cerebral blood flow (CBF) (22, 23). On the other hand, three studies found no such critical adverse events particularly no increase in ICP, when patients with acute brain injury were switched to isoflurane mono sedation (24–26). However, the number of patients in these studies were limited, duration of treatment was confined just to several hours, and patient’s sedation was switched to an isoflurane monotherapy, thereby causing a high incidence of potentially detrimental side effects like MAP and CPP reduction. Consequently, there is a lack of evidence concerning the safety and feasibility of long-term volatile sedation in patients with SAH.

However, some patients with acute severe aneurysmal SAH display signs of inadequate sedation depths, often times resulting in ventilator asynchrony, patient distress and agitation despite the use of high doses of conventional anesthetics. This inadequate sedation bears the risk of an increased CMRO_2_ and increased CBF and thereby potentially leads to brain edema formation and intracranial hypertension due to imbalances in cerebral oxygen delivery (4). The use of volatile anesthetics can be considered on an individual basis to achieve adequate sedation according to the *German evidence and consensus based guideline for the management of delirium, analgesia, and sedation in intensive care medicine* published in 2015 (2). Therefore, at our NICU, patients received low-dose volatile isoflurane in addition to the in-house standard of intravenous hypnotic drugs as a therapeutic attempt if deemed necessary to achieve a sufficient sedation depth according to the treating physician’s opinion.

In this retrospective study, we analyzed data obtained from our patient data management system (PDMS) enclosing a homogenous cohort of 36 patients with aneurysmal SAH in the NICU receiving a balanced sedation including volatile isoflurane in order to improve inadequately shallow sedation. Here, we report the immediate, short, medium and long-term effects by retrospectively analyzing data in the selected period from 48 h before and up to 144 h after the initiation of isoflurane on neuromonitoring data such as ICP, as well as hemodynamic, respiratory parameters, and laboratory measurements.

## 2. Methods

### 2.1. Patients

Ethical approval for this study (project number 20–006) was provided by the Ethical Committee of the Ludwig Maximilian University of Munich on 24 February 2020. The need for written informed consent was waived due the retrospective nature of the study in which all data were processed anonymously using standardized data query. Patient data were collected from the hospital’s patient data management system (PDMS) Q-Care ICU (Health Information Management GmbH, Bad Homburg, Germany).

All patients with the main diagnosis of aneurysmal subarachnoid hemorrhage who were hospitalized between August 2015 and November 2018 were identified. In a next step, all patients who had received volatile isoflurane sedation during the course of their ICU treatment were selected for further analysis. Initiation of volatile sedation was left to the discretion of the attending physician when insufficient sedation depth was deemed harmful for the patient’s outcome: insufficient depth of sedation as indicated by the Richmond Agitation Sedation Scale (RASS) > −5 or, if implemented, bispectral index monitoring >40 (Infinity^®^ Delta, BISx^™^ SmartPod, VF 8.4 software, Dräger AG, Lübeck, Germany) despite upper limit doses of intravenous sedatives due to our in-house sedation protocol. However, the following criteria had to be fulfilled before the initiation of additional volatile sedation: Mechanical ventilation, estimated sedation time > 5 days, and external ventricular drainage for ICP measurement in place. Following patients were not seen as suitable for volatile sedation and did not receive volatile sedation: Age < 18 years, ICP elevation above 20 mmHg for more than 5 min in the previous 48 h, pre-existing impaired pulmonary function (e.g., known COPD, partial arterial pressure CO_2_ (*P*aCO_2_) > 45 mmHg, *P*aO_2_/FiO_2_ < 200, positive end-expiratory pressure (PEEP) > 10 cmH_2_O, peak inspiratory pressure > 30 cmH_2_O), known history or family history of malignant hyperthermia.

### 2.2. Mechanical ventilation and sedation management

According to our in-house treatment guidelines, respiratory settings according to the guidelines for lung protective ventilation on www.ardsnet.org (mechanical ventilation protocol summary: tidal volume ≤ 6 mL/kg predicted body weight, using the “lower PEEP/higher FiO_2_” table) were chosen and settings were adjusted to target values by regular arterial blood gas analysis (*P*aO_2_ 70–100 mmHg, *P*aCO_2_ 35–45 mmHg, pH 7.35–7.45). Ventilator settings were documented each time a blood gas analysis was performed (at least four times in 24 h).

Patients were sedated according to an in-house standard protocol with continuous infusion of midazolam (0.15–0.7 mg/kg/h) ± propofol (1–4 mg/kg/h) ± ketamine (100–200 mg/h) combined with the analgesic opioid sufentanil (0.5–0.7 μg/kg/h). Volatile sedation was started with the Sedaconda^®^ anesthetic conserving device (ACD-L with dead space of 100 mL) by adding 2–6 mL/h of the pharmaceutical Sedaconda^®^ (isoflurane, both from Sedana Medical AB, Danderyd, Sweden) per inhalation. The administration of isoflurane via the medical device Sedacona^®^ has been approved for inhaled sedation in intensive care without any restriction in duration of therapy by the German pharmaceutical authority BfArM (Bonn, Germany). The dose of inhalational isoflurane was titrated to reach a target RASS of −5 and in case BIS monitoring was established, to establish an bispectral index of 20–40. Expiratory target concentration of 0.2–0.6 vol.% isoflurane was measured by an external gas monitoring device (Vamos^®^, Dräger AG, Lübeck, Germany). Minimal alveolar concentration (MAC) for a given age was calculated using the following formula: MAC_age_ = MAC_40_ × ^10–0.00269(age−40)^ (27).

Isoflurane administration was ceased when need of deep sedation was no longer required. Since the use of volatile anesthetics is contraindicated in patients with increased ICP according to the *German evidence and consensus based guideline* (2) we stopped the administration of isoflurane immediately when ICP was >20 mmHg for more than 5 min.

All patients were screened for other potential severe adverse events (malignant hyperthermia, liver and renal impairment, and ventilation impairment). Therefore, serum liver enzymes, renal functional parameters and markers for acute rhabdomyolysis were tested on a daily basis. In case of a supposed adverse event, the treating physician terminated volatile sedation by individual decision.

### 2.3. SAH management

SAH treatment followed standardized management protocols. Patients underwent surgical aneurysmal clipping or interventional neuroradiologic coiling as decided by a joint neurovascular committee during the first 24 h following hospital admission. NICU treatment included continuous ICP measurement via an external ventricular drainage (EVD, Integra Life Sciences, Ratingen, Germany). As zero reference point for the EVD transducer we use the external acoustic channel; for measuring invasive blood pressure we used the level of the phlebostatic axis as the optimal measurement location remains debated (28, 29). The EVD was left open to drainage cerebral spinal fluid above a individually defined pressure threshold. The EVD was closed hourly by the nursing staff in order to assess the intracranial pressure. When the ICP was above 20 mmHg for more than 5 min the attending physician was informed. ICP management followed a step-wise protocol including balanced sedation, arterial blood gas homeostasis, targeted CPP management, hypertonic fluids, and surgical craniotomies as described elsewhere (30) in accordance with current guidelines (31, 32).

Patients were neurologically examined twice daily by the treating physician (pupillary reaction, reflex status, muscle tone), pupillary reaction and Glasgow Coma Score (GCS) were additionally checked every hour by the nursing staff. Occurrence of cerebral vasospasm was assessed by daily transcranial Doppler sonography. Cerebral imaging was performed depending on the patient’s clinical course. All relevant patient parameters (ICP, MAP, CPP, heart rate, urinary bladder temperature, and laboratory results) were documented via a direct interface with the PDMS. The nursing staff of the NICU validated all hourly measured values and checked for potential measurement errors before the data were transferred to the PDMS.

### 2.4. Statistical analysis

All data were collected from the NICU PDMS. To investigate immediate, short- mid-, and long-term changes (see below for definition), we calculated the means ± standard deviation (SD) of all documented values available during the respective period for each patient and compared the mean values of all patients obtained before the initiation of isoflurane with the means after the start of therapy. Immediate: One hour before to 1 hour after the initiation of isoflurane. Short-term: -period 6 h to -1 h before isoflurane vs. period +1 h to +6 h after isoflurane, mid-term: −24 h to −1 h vs. +1 to 24 h, long-term: −48 h to −1 h vs. +1 h to +144 h. As cerebral pathomechanisms in SAH vary over time we focused on the actual effects on the induction of balanced volatile sedation during the initial treatment phase on NICU. Therefore, we decided to end the retrospective data analysis after 144 h (6 days), although most patients received isoflurane for a longer period. If fewer than four values were documented in the above-mentioned periods, the patient was excluded from the respective parameter analysis. The number of patients included in the analysis at any time point is given for all investigated parameters. For categorical data, absolute and relative frequencies are given. For statistical analysis, the differences of each patient between the parameters before and after administration of isoflurane were tested for normality using the Shapiro–Wilk or D’Agostino-Pearson test. Normally distributed data were examined with a paired Student’s t-test, non-normally distributed data with Wilcoxon matched-pairs signed-ranks Test. Comparison of baseline parameters (period -24 h to -1 h) between patients with or without following premature termination of volatile sedation was performed using a unpaired Student’s *t*-test or Mann–Whitney *U*-test. We did not adjust for multiple comparisons; thus, all *value of p*s are descriptive. *p* -values <0.05 were considered as significant. We collected data using Microsoft Excel 2016 and performed statistical evaluation using Prism version 7.2 (Graph Pad Software, San Diego, CA, United States).

## 3. Results

### 3.1. Patient demographics and outcome

One hundred and twelve patients were admitted to the NICU of the University Hospital of LMU Munich with the primary diagnosis of aneurysmal SAH between 08/2015 and 11/2018. Thirty-six patients received balanced volatile sedation with isoflurane via the Sedaconda^®^ ACD. The overall mortality in the NICU was generally low, reaching 2.77% (1 out of 36 patients) given the recorded SAH severity with a median of Fisher 4 [interquartile range (IQR, 4 to 4) and WFNS 4 (IQR, 2 to 5)] (Table 1). Volatile sedation was established on average 3 days [IQR, 2 to 5 days] after admission to the NICU. Patients received isoflurane for mean period of 9.73 ± 7.56 days at a dose of 2–6 mL/h (mean 2.74 ± 0.71 mL/h). This corresponded to an expiratory concentration of 0.2–0.6 vol.% and a minimal alveolar concentration (MAC) of approximately 0.19–0.57 for a 56-year old patient (mean age of the cohort).

**Table 1:** Patient’s baseline demographic and clinical parameters.

	No.	Age (years)	Sex	Start isoflurane (days after admission)	Indication for discontinued isoflurane	Duration volatile sedation (days)	SAH fischer grade	SAH WFNS grade	Intervention treatment	ICU LOS (days)	Death on ICU
No clinical indication for termination (*n* = 27, 75%)	1	73	Male	5	–	5	4	V	Coil	29	0
	2	76	Female	3	–	7	4	V	Coil	40	0
	3	53	Male	3	-	17	3	I	Clip + Coil	32	0
	4	41	Male	2	–	21	3	IV	Coil	32	0
	5	55	Female	5	–	14	4	II	Coil	27	0
	6	71	Female	2	–	4	4	IV	Clip	26	0
	7	57	Female	1	–	20	4	V	Clip	32	0
	8	58	Female	7	–	13	2	IV	Clip + Coil	17	0
	9	46	Female	2	-	13	3	II	Clip	20	0
	10	80	Female	5	–	5	3	II	Coil	26	0
	11	49	Female	3	–	30	4	IV	Clip + Coil	62	0
	12	37	Female	5	–	6	3	III	Coil	28	0
	13	64	Male	3	–	4	4	V	Coil	26	0
	14	62	Female	9	–	9	4	IV	Clip	26	0
	15	66	Female	12	–	10	4	I	Coil	36	0
	16	50	Female	2	–	4	4	III	Coil + Clip	36	0
	17	52	Male	2	–	21	4	V	FlowDiver.	64	0
	18	63	Female	2	–	12	4	V	Clip	28	0
	19	57	Female	1	–	15	4	V	Coil	34	0
	20	65	Female	5	–	4	4	V	Clip	28	0
	21	51	Female	2	–	22	4	V	Clip	37	0
	22	51	Female	5	–	17	3	II	Clip	40	0
	23	50	Male	1	–	4	4	II	No	40	0
	24	47	Female	2	–	21	3	II	Clip	32	0
	25	45	Female	13	–	12	4	V	Clip	33	0
	26	41	Male	9	–	12	4	II	FlowDiver.	31	0
	27	53	Female	10	–	7	4	V	Coil/WebDev.	16	1
Indicated termination of volatile sedation (*n* = 9, 25%)	28	53	Female	11	ICP/LEE	<1 h/5d	4	V	Clip	43	0
	29	55	Female	4	CO_2_	33 h	4	V	Coil	31	0
	30	70	Female	15	ICP	14 h	4	IV	Clip	35	0
	31	53	Female	3	ICP	30 h	4	V	Coil	58	0
	32	57	Female	2	CO_2_	20 h	4	II	Clip	33	0
	33	49	Female	1	ICP	4	4	V	Coil	26	0
	34	45	Male	0	CO_2_	11 h	4	IV	Clip	46	0
	35	51	Male	5	ICP	6	4	IV	Clip	65	0
	36	51	Male	2	CO_2_	39 h	4	III	Clip	35	0
**Overall cohort**		**56 (10.1)**	**10/26 (m/f)**	**3 [2 to 5]**	**5/4 (ICP/CO2)**	**9.73 (7.56)**	**4 [4 to 4]**	**4 [2 to 5]**		**32 [27 to 39]**	**2.77%**

SAH, subarachnoid hemorrhage; ICP, intracranial pressure crisis; LEE, critical liver enzyme elevation; CO2, refractory hypercapnia; Fisher Grade; WFNS grade, World Federation of Neurological Surgeons; ICU, intensive care unit; LOS, length of stay; Interventions/treatment: Clip, aneurysmal clipping; Coil, aneurysmal coiling; FlowDiv., aneurysmal treatment via intraluminal flow diverter; WebDev., aneurysmal treatment via intraluminal web device; Values given for the overall cohort are means (±SD) or median (IQR) as not stated otherwise.

### 3.2. Immediate and short-term changes

**Figure 1:**
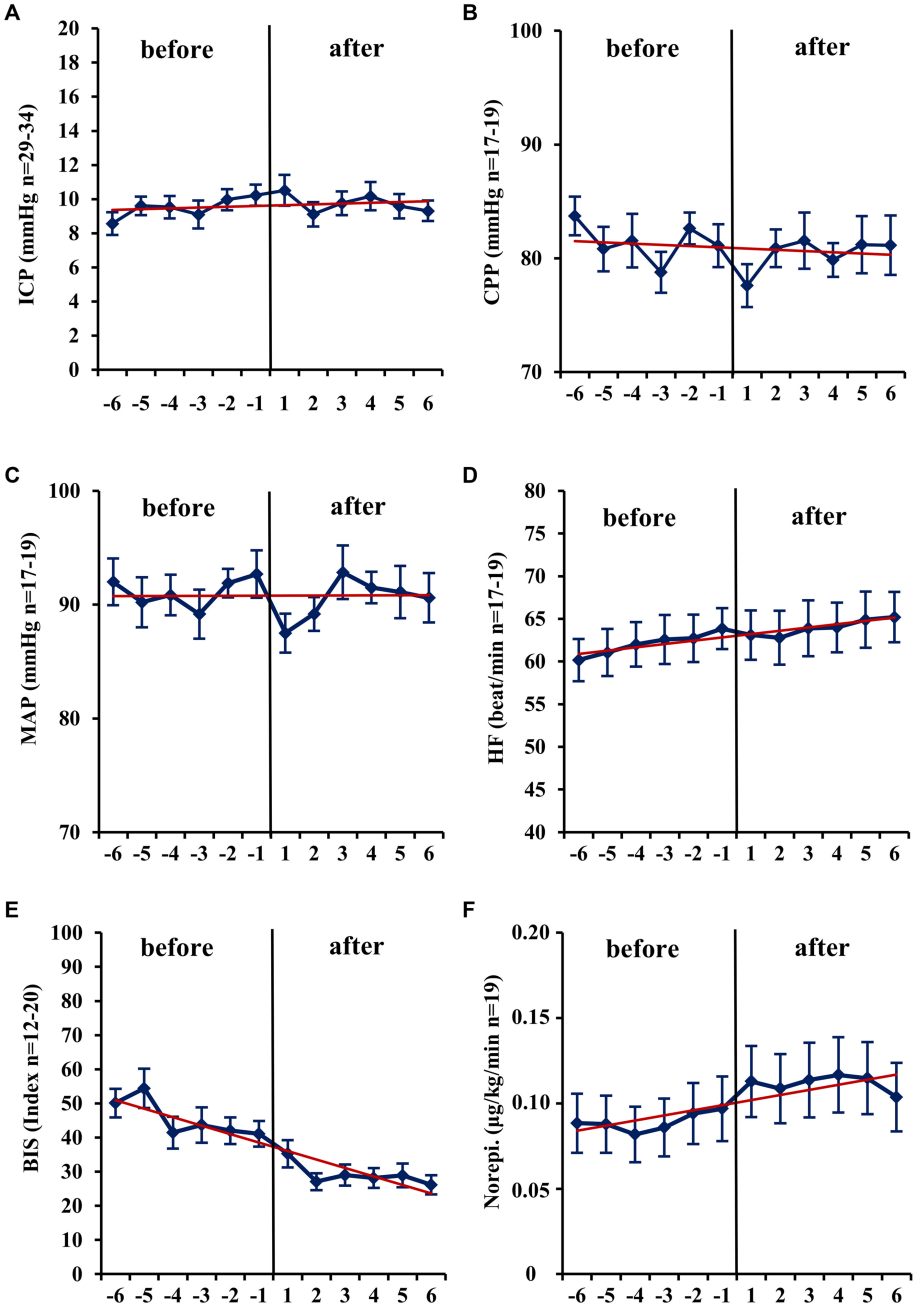
Short-term changes (− 6 h to +6 h) before and after administration of volatile isoflurane over time. ICP, intracranial pressure **(A)**; CPP, cerebral perfusion pressure **(B)**; MAP, mean arterial pressure **(C)**; HF, heart frequency **(D)**; BIS, bispectral index **(E)**; Norepi., Norephinephrine dose **(F)**; n, number of patients with available data. Data represents the hourly means (−6 h to +6 h) of all patients with available data at that time. Error bars given are SEM, standard error of the mean. The trend line shown is calculated as linear regression.

Within the first hour after the initiation of isoflurane, the mean ICP increased only slightly (−1 h: 10.32 vs. +1 h: 10.5_2_ mmHg; *n* = 31; *p* = 0.671; Table 2; Figure 1). In contrast, MAP (−4.67 mmHg; *n* = 18; *p* = 0.014) and, consequently, CPP (−4.21 mmHg; *n* = 14; *p* = 0.013) decreased significantly. Therefore, a significant increase in vasopressor dosage (norepinephrine +0.022 μg/kg/min; *n* = 19; *p* = 0.039) was necessary in order to compensate for the decrease in MAP and CPP. In none of the patients a critical CPP of <60 mmHg was observed (minimum 61 mmHg). In the short-term analysis, which includes the means of hourly values from 6 h to 1 h before and 1 h to 6 h after initiation of volatile sedation, CPP and MAP rose compared with values measured at the time point -1 h (Table 2 and Figure 1) indicating hemodynamic stabilization. However, this was achieved by keeping the norepinephrine dosages significantly increased compared with the pre-isoflurane baseline. ICP did not significantly change in the short-term analysis, although there was a slight mean increase (6 h to 1 h before versus +1 h to +6 h after start of isoflurane +0.34 mmHg; *n* = 36; *p* = 0.443). Consistent with the hypothesis that additional volatile sedation would result in a deeper sedation level BIS monitoring showed significantly decreased values after isoflurane was established (−15.16; *n* = 16; *p* = 0.005; −6 h to −1 h before versus +1 h to +6 h after isoflurane).

**Table 2:** Immediate and short term changes in intracranial pressure and systemic vital parameters before and after additional sedation with volatile isoflurane.

	Parameter	Before isoflurane (−1 h)	After isofluran (+1 h)	Difference	95% CI	*n*	*p*-value
Cerebral monitoring	ICP (mmHg)	10.23 (3.43)	10.52 (5.07)	0.29 (3.77)	−1.09 to 1.67	31	0.671
	CPP (mmHg)	81.29 (8.19)	77.07 (8.05)	−4.21 (5.45)	−7.36 to −1.07	14	0.013
	BIS (Index)	39.14 (15.81)	35.00 (15.98)	−4.14 (13.70)	−12.06 to 3.77	14	0.222
Cardiovascular system	HF (beats/min)	64.78 (9.89)	64.72 (12.69)	−0.06 (6.51)	−3.29 to 3.18	18	0.972
	MAP (mmHg)	92.78 (9.33)	88.11 (7.05)	−4.67 (8.23)	−8.76 to −0.58	18	0.014
	Temp (°C)	36.86 (0.77)	36.85 (0.69)	−0.006 (0.170)	−0.090 to 0.079	18	0.891
	Norepi. (μg/kg/min)	0.094 (0.078)	0.117 (0.094)	0.022 (0.043)	−0.002 to 0.043	19	0.039

	Parameter	Before isoflurane (−6 h to −1 h)	After isoflurane (+1 to + 6 h)	Difference	95% CI	*n*	*p*-value
Cerebral monitoring	ICP (mmHg)	9.33 (3.07)	9.66 (3.29)	0.34 (2.62)	−0.55 to 1.22	36	0.443
	CPP (mmHg)	82.40 (6.64)	80.20 (5.50)	−2.23 (5.49)	−4.96 to 0.50	18	0.103
	BIS (Index)	42.08 (15.05)	26.91 (12.13)	−15.16 (18.66)	−25.11 to −5.22	16	0.006
Cardiovascular system	HF (beats/min)	63.70 (10.91)	64.73 (12.75)	1.03 (6.32)	−2.02 to 4.08	19	0.487
	MAP (mmHg)	91.30 (6.63)	90.19 (5.30)	−1.10 (5.19)	−3.60 to 1.40	19	0.366
	Temp (°C)	36.96 (0.69)	36.76 (0.70)	−0.21 (0.59)	−0.49 to 0.08	19	0.166
	Norepi. (μg/kg/min)	0.085 (0.067)	0.115 (0.092)	0.030 (0.055)	0.003 to 0.056	19	0.029

ICP, intracranial pressure; CPP, cerebral perfusion pressure; BIS, bispectral index; HF, heart frequency; MAP, mean arterial pressure; Temp, body core temperature; Norepi., norepinephrine dose, 95% CI, 95% confidence interval of the mean; n, number of patients with available data. Data represents the means (±SD) of immediate (time points −1 h vs + 1 h, values documented at this time) and short-term (means of period -6 h to −1 h vs + 1 h to + 6 h), changes before and after additional sedation with volatile isoflurane.

### 3.3. Mid- and long-term changes

**Figure 2:**
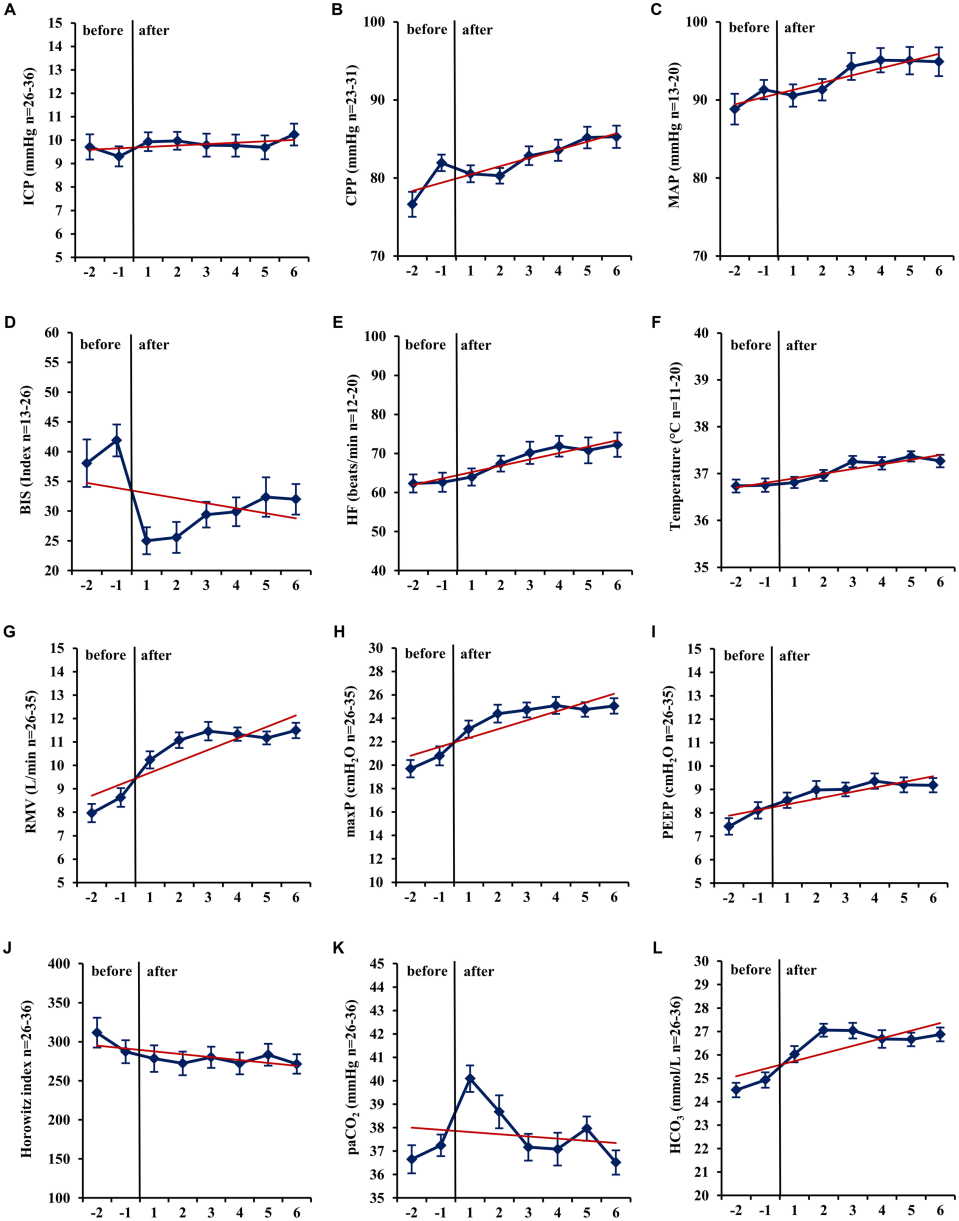
Long-term changes before and after administration of volatile isoflurane over time (−2 days to +6 days). ICP, intracranial pressure **(A)**; CPP, cerebral perfusion pressure **(B)**; MAP, mean arterial pressure **(C)**; BIS, bispectral index **(D)**; HF, heart frequency **(E)**; Temp, body core temperature **(F)**; RMV, respiratory minute volume **(G)**; maxP, peak inspiratory pressure **(H)**; PEEP, post endexpiratory pressure **(I)**; Horowitz index, *P*aO_2_/FiO_2_, *P*aO_2_, arterial partial pressure of oxygen **(J)**; *P*aCO_2_, arterial partial pressure of carbon dioxide **(K)**; HCO_3−_, standard bicarbonate **(L)**; *n*, number of patients with available data. Data represent the daily means (−2 days to +6 days) of all patients with available data at that time. The error bars represent the SEM, standard error of the mean. The trend line shown was calculated using linear regression.

In mid-term (−24 h to −1 h vs. +1 h to +24 h; Table 3) and long-term analysis (−48 h to −1 h vs. +1 to +144 h; Figure 2 and Table 4), a non-significant increase in ICP (mid-term: +0.60 mmHg, *n* = 36, *p* = 0.057; long-term: +0.65 mmHg, *n* = 36, *p* = 0.055) was observed. In the period from +1 h to +24 h after initiation of the ACD patients needed a significantly higher ventilator minute volume (+1.64 L/min; *n* = 33; *p* < 0.001), an increased peak inspiratory pressure (+2.26 cmH_2_O; *n* = 33; *p* < 0.001) and PEEP (+0.45 cmH_2_O; *n* = 33; *p* = 0.037, max. 15 cmH_2_O) to maintain an adequate *P*aCO_2_ when compared with the 24 h period before initiation of isoflurane. Despite adjustment to more invasive ventilation settings, *P*aCO_2_ increased significantly to a mean of 40.14 mmHg (+2.90 mmHg; *n* = 36; *p* < 0.001; −24 to -1 h vs. +1 to +24 h). An analysis of blood markers revealed significantly higher concentrations of liver serum parameters, markers of muscle cell damage, and serum urea in the long-term period before vs. after isoflurane sedation. Compared with the period before volatile sedation (−48 h to −1 h) patients received less intravenous propofol sedation after the addition of isoflurane without reaching a significant threshold (−51.03 mg/h; *n* = 18; *p* = 0.077).

**Table 3:** Mid-term changes (− 24 h to +24 h) before and after administration of volatile isoflurane.

	Parameter	Before isoflurane (−24 h to −1 h)	After isoflurane (+1 h to + 24 h)	Difference	95% CI	*n*	*p*-value
Cerebral monitoring	ICP (mmHg)	9.34 (2.61)	9.95 (2.44)	0.60 (1.84)	−0.02 to 1.23	36	0.057
	CPP (mmHg)	81.61 (5.63)	80.56 (6.35)	−1.05 (5.12)	−3.08 to 0.97	27	0.295
	BIS (Index)	41.63 (12.76)	26.11 (12.61)	−15.52 (12.41)	−21.17 to −9.87	21	<0.001
Cardiovascular system	HF (beats/min)	63.56 (11.37)	64.90 (10.19)	1.34 (5.30)	−1.22 to 3.89	19	0.287
	MAP (mmHg)	91.62 (5.59)	90.77 (6.83)	−0.85 (4.37)	−2.95 to 1.26	19	0.410
	Temp (°C)	36.72 (0.62)	36.74 (0.50)	0.02 (0.48)	−0.21 to 0.25	19	0.847
Respiratory function	SaO_2 (%)_	98.06 (1.39)	97.67 (1.20)	−0.39 (0.98)	−0.86 to 0.08	19	0.102
	FiO_2_	38.08 (7.09)	40.93 (8.89)	2.86 (6.28)	0.70 to 5.01	35	0.032
	RMV (L/min)	8.66 (2.14)	10.29 (1.85)	1.64 (1.33)	1.17 to 2.12	33	<0.001
	Compliance	55.97 (12.68)	56.51 (13.25)	0.54 (7.08)	−1.97 to 3.05	33	0.905
	maxP (cmH_2_O)	20.90 (4.36)	23.16 (3.83)	2.26 (2.12)	1.51 to 3.01	33	<0.001
	PEEP (cmH_2_O)	8.14 (1.88)	8.59 (1.73)	0.45 (1.22)	0.02 to 0.88	33	0.037
	Horowitz index	288.10 (73.27)	274.70 (83.13)	−13.43 (43.32)	−28.31 to 1.46	35	0.076
	paO2 (mmHg)	105.60 (17.06)	105.60 (16.21)	−0.09 (18.65)	−6.41 to 6.21	36	0.786
Acid–base homeostasis	pH	7.43 (0.04)	7.43 (0.04)	−0.005 (0.04)	−0.02 to 0.01	36	0.509
	paCO2 (mmHg)	37.24 (2.64)	40.15 (3.29)	2.90 (4.28)	1.47 to 4.36	36	<0.001
	HCO_3_^−^ (mmol/L)	24.93 (1.83)	26.04 (1.96)	1.11 (1.63)	0.56 to 1.66	36	<0.001
	Lactate (mmol/L)	1.40 (0.69)	1.41 (0.57)	0.01 (0.53)	−0.17 to 0.19	36	0.913
Drugs	Isoflurane (mL/h)	0 (0)	2.61 (0.91)	2.61 (0.91)	2.14 to 3.08	17	<0.001
	Ketamine (mg/h)	103.40 (85.45)	129.30 (77.24)	25.83 (60.39)	−5.21 to 56.88	17	0.097
	Midazolam (mg/h)	21.29 (20.39)	23.79 (19.32)	2.51 (12.49)	−3.92 to 8.93	17	0.600
	Nimodipin (mg/h)	0.74 (0.64)	0.71 (0.74)	−0.03 (0.62)	−0.35 to 0.29	17	0.847
	Norepi. (μg/kg/min)	0.084 (0.062)	0.118 (0.086)	0.034 (0.059)	0.005 to 0.062	19	0.024
	Sufentanil (μg/h)	35.44 (20.78)	30.63 (16.53)	−4.81 (15.73)	−13.52 to 3.91	15	0.194
	Propofol (mg/h)	72.00 (128.2)	27.63 (40.63)	−44.36 (123.2)	−107.70 to 18.99	17	0.432
Serum parameters	ALP (U/L)	186.10 (193.6)	203.90 (184.2)	17.79 (53.04)	−17.84 to 53.42	11	0.292
	Bilirubine (mg/dL)	0.43 (0.16)	0.38 (0.16)	−0.05 (0.13)	−0.106 to 0.010	23	0.102
	CK (U/)l	324.80 (448.2)	428.00 (520.4)	103.30 (256.2)	−110.9 to 317.4	8	0.641
	CRP (mg/dL)	5.43 (4.09)	5.59 (3.61)	0.16 (2.96)	−0.84 to 1.16	36	0.945
	GGT (U/L)	271.30 (449.4)	311.10 (452.9)	39.79 (138.7)	−9.38 to 88.96	33	0.003
	ALT (U/L)	55.46 (65.44)	53.79 (62.93)	−1.68 (37.81)	−16.34 to 12.98	28	0.933
	AST (U/L)	65.06 (96.22)	67.94 (85.5)	2.88 (33.52)	−9.01 to 14.76	33	0.453
	Urea (mg/dL)	29.68 (13.62)	33.62 (15.91)	3.93 (6.02)	1.87 to 6.00	35	<0.001
	IL-6 (pg/dL)	30.29 (26.73)	23.31 (16.97)	−6.98 (22.93)	−14.74 to 0.78	36	0.105
	Creatinine (mg/dL)	0.82 (0.22)	0.84 (0.25)	0.03 (0.08)	−0.002 to 0.054	35	0.090
	Myoglobine (mg/dL)	125.80 (147.80)	258.90 (465.4)	133.10 (407.90)	−16.56 to 282.70	31	0.490

ICP, intracranial pressure; CPP, cerebral perfusion pressure; BIS, bispectral index; HF, heart frequency; MAP, mean arterial pressure; Temp, body core temperature; SaO_2_, peripheral oxygen saturation; FiO_2_, fraction of inspired oxygen; RMV, respiratory minute volume; maxP, peak inspiratory pressure; PEEP, post-end-expiratory pressure, Horowitz index, PaO_2_/FiO_2_; PaO_2_, arterial partial pressure of oxygen; PaCO_2_, arterial partial pressure of carbon dioxide; pH, blood pH in blood gas analysis; HCO_3−_, standard bicarbonate; Norepi., norepinephrine dose; Serum parameters: ALP, alkaline phosphatase; CK, creatine kinase; CRP, C-reactive protein; GGT, gamma glutamyl transpeptidase; AST, aspartate transaminase; ALT, alanine transaminase; IL-6, interleukin 6; 95% CI, 95% confidence interval of the mean; n, number of patients with available data. Data represent means (±SD) or median (IQR) of all values given in the period (−24 h to −1 h vs + 1 h to + 24 h) before and after additional sedation with volatile isoflurane.

**Table 4:** Long-term changes (− 48 h to +144 h) before and after administration of volatile isoflurane.

	Parameter	Before isoflurane (−48 h to −1 h)	After isoflurane (+1 h to + 144 h)	Difference	95% CI	*n*	*p*-value
Cerebral monitoring	ICP (mmHg)	9.48 (2.49)	10.13 (2.15)	0.65 (1.98)	−0.02 to 1.32	36	0.055
	CPP (mmHg)	79.29 (7.10)	81.95 (7.13)	2.67 (6.97)	−0.69 to 6.03	19	0.196
	BIS (Index)	41.63 (15.54)	24.76 (10.29)	−16.87 (14.33)	−26.50 to −7.24	11	<0.001
Cardiovascular system	HF (beats/min)	64.06 (10.99)	68.48 (10.02)	4.42 (5.67)	1.69 to 7.15	19	0.003
	MAP (mmHg)	90.25 (6.38)	92.77 (7.26)	2.51 (5.43)	−0.10 to 5.13	19	0.096
	Temp (°C)	36.68 (0.55)	36.96 (0.49)	0.28 (0.39)	0.09 to 0.48	19	0.005
Respiratory function	SaO_2 (%)_	98.03 (1.26)	97.98 (0.73)	−0.04 (1.19)	−0.61 to 0.53	19	0.145
	FiO_2_	38.07 (7.25)	41.51 (8.27)	3.44 (7.46)	0.89 to 6.01	35	0.015
	RMV (L/min)	8.31 (2.03)	11.01 (1.58)	2.71 (1.36)	2.22 to 3.19	33	<0.001
	Compliance	56.62 (12.60)	55.70 (11.51)	−0.92 (7.15)	−3.45 to 1.62	33	0.469
	maxP (cmH_2_O)	20.31 (4.03)	24.43 (3.37)	4.14 (2.79)	3.14 to 5.13	33	<0.001
	PEEP (cmH_2_O)	7.81 (1.78)	8.88 (1.61)	1.07 (1.52)	0.53 to 1.61	33	<0.001
	Horowitz index	302.00 (84.17)	282.80 (65.4)	−19.19 (47.93)	−37.42 to −0.96	29	0.040
	paO2 (mmHg)	110.60 (18.92)	107.30 (12.81)	−3.34 (18.1)	−9.47 to 2.78	36	0.276
Acid–base homeostasis	pH	7.426 (0.033)	7.451 (0.026)	0.025 (0.040)	0.011 to 0.038	36	<0.001
	paCO2 (mmHg)	37.14 (2.56)	38.37 (2.74)	1.23 (3.44)	0.07 to 2.40	36	0.039
	HCO_3_^−^ (mmol/L)	24.61 (1.56)	26.67 (1.30)	2.06 (1.87)	1.43 to 2.69	36	<0.001
	Lactate (mmol/L)	1.39 (0.67)	1.17 (0.30)	−0.22 (0.54)	−0.40 to −0.03	36	0.022
Drugs	Isoflurane (mL/h)	0.00 (0.)	2.74 (0.71)	2.74 (0.71)	2.39 to 3.09	18	<0.001
	Ketamine (mg/h)	90.03 (85.83)	117.90 (59.49)	27.876 (79.71)	−11.77 to 67.51	18	0.156
	Midazolam (mg/h)	18.07 (18.62)	18.59 (14.73)	0.52 (14.43)	−6.66 to 7.70	18	0.881
	Nimodipin (mg/h)	0.66 (0.59)	0.75 (0.64)	0.10 (0.75)	−0.28 to 0.47	18	0.596
	Norepi. (μg/kg/min)	0.084 (0.061)	0.098 (0.060)	0.014 (0.047)	−0.009 to 0.036	18	0.220
	Sufentanil (μg/h)	34.27 (18.00)	23.69 (14.48)	−10.58 (16.15)	−18.61 to −2.55	18	0.002
	Propofol (mg/h)	72.36 (103.4)	21.33 (36.19)	−51.03 (104.40)	−103 to 0.90	18	0.077
Serum parameters	ALP (U/L)	107.40 (105.6)	163.80 (151.3)	56.44 (72.13)	23.60 to 89.27	21	<0.001
	Bilirubine (mg/dL)	0.55 (0.27)	0.45 (0.16)	−0.10 (0.25)	−0.20 to 0.004	25	0.057
	CK (U/L)	182.20 (264.3)	257.80 (326.3)	75.61 (264.7)	−48.26 to 199.5	20	0.047
	CRP (mg/dL)	4.70 (3.92)	7.61 (4.64)	2.91 (6.08)	0.75 to 5.07	33	0.005
	GGT (U/L)	104.70 (157.4)	312.50 (404.9)	207.80 (304.5)	91.95 to 323.60	29	<0.001
	ALT (U/L)	36.19 (25.12)	59.68 (54.98)	23.49 (45.61)	6.46 to 40.52	30	0.004
	AST (U/L)	37.21 (27.89)	65.90 (68.67)	28.69 (53.22)	9.51 to 47.88	32	<0.001
	Urea (mg/dL)	28.53 (10.92)	44.88 (17.08)	16.35 (16.67)	10.63 to 22.08	35	<0.001
	IL-6 (pg/dL)	27.58 (20.07)	53.28 (55.10)	25.70 (61.88)	4.44 to 46.96	35	0.011
	Creatinine (mg/dL)	0.81 (0.21)	0.85 (0.27)	0.03 (0.13)	−0.01 to 0.07	35	0.211
	Myoglobine (mg/dL)	110.50 (141.2)	282.80 (294.4)	172.30 (225.0)	88.30 to 256.30	30	<0.001

ICP, intracranial pressure; CPP, cerebral perfusion pressure; BIS, bispectral index; HF, heart frequency; MAP, mean arterial pressure; Temp, body core temperature; SaO_2_, peripheral oxygen saturation; FiO_2_, fraction of inspired oxygen; RMV, respiratory minute volume; maxP, peak inspiratory pressure; PEEP, post-end-expiratory pressure, Horowitz index, PaO_2_/FiO_2_; PaO_2_, arterial partial pressure of oxygen; PaCO_2_, arterial partial pressure of carbon dioxide; pH, blood pH in blood gas analysis; HCO_3−_, standard bicarbonate; Norepi., norepinephrine dose; Serum parameters: ALP, alkaline phosphatase; CK, creatine kinase; CRP, C-reactive protein; GGT, gamma glutamyl transpeptidase; AST, aspartate transaminase; ALT, alanine transaminase; IL-6, interleukin 6; 95% CI, 95% confidence interval of the mean; n, number of patients with available data. Data represent means (±SD) or median (IQR) of all values given in the period (−48 h to −1 h vs + 1 h to + 144 h) before and after additional sedation with volatile isoflurane.

### 3.4. Early termination of isoflurane/adverse reactions

In 9 out of 36 patients, isoflurane sedation was stopped prematurely due to potential adverse side effects even though increased administration of anesthetics was considered necessary to maintain adequate sedation depth. Mainly, those patients exhibited ICP increases (5 patients with a mean ICP of 27.20 ± 6.54 mmHg, maximum of 37 mmHg after a median of 30 h [IQR, 7.5 to 125.5] following isoflurane administration). In one patient, the volatile sedation was terminated after 10 min isoflurane initiation due to an ICP peak (25 mmHg). Of note, the patient in question received isoflurane at a later treatment stage (starting on day 11 in the NICU) without developing an acute increase of ICP in the following 5-day period. However, this patient displayed critically increased liver serum parameters (γ-GT 2239 U/L, AST 693 U/L, ALT 1075 U/L) and hence isoflurane administration was discontinued again. The second most common reason for premature termination of volatile sedation was respiratory hypercapnic insufficiency with elevated *P*aCO_2,_ which was not controllable by increasing ventilation parameters (4 patients, with a mean *P*aCO_2_ 57.20 ± 7.57 mmHg after a median 26.5 h [IQR, 13.25 to 37.5]).

It is noteworthy that the subgroup of patients with prematurely terminated isoflurane, and in particular those experiencing an elevated *P*aCO_2,_ had a significantly higher peak inspiratory pressure, lower lung compliance, and showed a trend towards higher ventilator minute volume in the period -24 h to -1 h prior to the implementation of the ACD when compared with the patients with continued isoflurane sedation (Table 5, Supplementary Table S1). None of the investigated patients met the diagnostic criteria for malignant hyperthermia, newly developed renal impairment or other severe side effects except those listed above.

**Table 5:** Comparison of baseline parameters between patients who tolerated volatile sedation and patients with premature termination of volatile sedation.

	Parameter	Continued volatile sedation *n* = 11–27 (−24 h to −1 h)	Discontinued volatile sedation *n* = 5–9 (−24 h to −1 h)	Difference	*p*-value
Baseline parameters	Age (years)	56.04 (11.00)	53.78 (7.00)	−2.26 (3.93)	0.673
	Sex (m/f)	7/20 (26/74%)	3/6 (33/66%)	–	–
	Duration (hours)	288 [120 to 408]	30 [12.5 to 67.5]	−258	<0.001
	Fisher scale	4 [3 to 4]	4 [4 to 4]	0	>0.999
	WFNS score	4 [2 to 5]	4 [3.5 to 5]	0	>0.999
Cerebral monitoring	ICP (mmHg)	9.42 (2.78)	9.11 (2.15)	−0.31 (1.02)	0.759
	CPP (mmHg)	80.69 (5.85)	84.22 (4.28)	3.53 (2.42)	0.158
	BIS (Index)	41.01 (9.11)	44.27 (25.14)	3.25 (7.24)	0.658
Cardiovascular system	HF (beats/min)	64.03 (12.48)	62.25 (8.54)	−1.78 (6.08)	0.964
	MAP (mmHg)	90.74 (5.65)	94.07 (5.15)	3.32 (2.89)	0.265
	Temp (°C)	36.90 (0.49)	36.22 (0.75)	−0.68 (0.29)	0.032
Respiratory function	SaO_2_ (%)	97.83 (1.45)	98.70 (1.10)	0.87 (0.72)	0.239
	FiO_2_	37.60 (6.92)	39.46 (7.81)	1.85 (2.76)	0.507
	RMV (L/min)	8.30 (1.5)	9.76 (3.37)	1.45 (0.84)	0.094
	Compliance	58.46 (11.99)	48.20 (12.3)	−10.25 (4.90)	0.045
	maxP (cmH_2_O)	19.80 (3.76)	24.32 (4.56)	4.52 (1.61)	0.009
	PEEP (cmH_2_O)	8.02 (1.92)	8.49 (1.79)	0.47 (0.77)	0.545
	Horowitz index	284.17 (73.74)	299.54 (75.00)	15.37 (28.6)	0.595
	paO_2_ (mmHg)	103.66 (17.41)	111.59 (15.34)	7.41 (6.52)	0.231
Acid–base homeostasis	pH	7.42 (0.03)	7.45 (0.04)	0.03 (0.014)	0.034
	paCO_2_ (mmHg)	37.66 (2.24)	35.97 (3.42)	−1.69 (0.99)	0.097
	HCO_3_^−^ (mmol/L)	24.73 (1.82)	25.54 (1.82)	0.81 (0.70)	0.254
	Lactate (mmol/L)	1.40 (0.73)	1.39 (0.60)	−0.01 (0.27)	0.823
Drugs	Ketamine (mg/h)	94.64 (90.39)	132.03 (69.5)	37.39 (49.50)	0.462
	Midazolam (mg/h)	17.17 (20.19)	34.67 (16.58)	17.50 (11.20)	0.144
	Nimodipin (mg/h)	0.84 (0.6)	0.41 (0.72)	−0.43 (0.36)	0.345
	Norepi. (μg/kg/min)	0.081 (0.069)	0.092 (0.042)	0.012 (0.033)	0.500
	Sufentanil (μg/h)	34.32 (23.81)	38.49 (10.38)	0.41 (12.5)	0.745
	Propofol (mg/h)	86.42 (142.58)	25.10 (50.21)	−61.32 (74.0)	0.336

M, male; f, female; duration, duration of volatile sedation; SAH, subarachnoid hemorrhage; Fisher Grade; WFNS grade, World Federation of Neurological Surgeons; ICP, intracranial pressure; CPP, cerebral perfusion pressure; BIS, bispectral index; HF, heart frequency; MAP, mean arterial pressure; Temp, body core temperature; SaO2, peripheral oxygen saturation; FiO2, fraction of inspired oxygen; RMV, respiratory minute volume; maxP, peak inspiratory pressure; PEEP, post endexpiratory pressure, Horowitz index, PaO_2_/FiO_2_; PaO_2_, arterial partial pressure of oxygen; PaCO_2_, arterial partial pressure of carbon dioxide; pH, blood pH in blood gas analysis; HCO3-, standard bicarbonate; Norepi., norepinephrine dose; n, number of patients with available data. Data represent means (±SD) or median (IQR) of all values given in the period (−24 h to −1 h before start of isoflurane).

## 4. Discussion

In the present retrospective analysis, we present data from 36 patients with severe aneurysmal SAH who received continuous isoflurane application in addition to a standard intravenous sedation regime in order to achieve an adequately deep sedation. While we measured positive effects on sedation depth by means of significantly decreased BIS values, we did not detect significant increases in ICP neither in the short-, mid-, nor long-term analysis (up to +144 h) in the vast majority of patients. However, in 25% of the patients balanced isoflurane sedation was stopped due ICP elevation (median 30 h after ACD implementation), or a clinically meaningful increase of PaCO2 or liver enzymes. This raises significant concerns about the safety of balanced sedation in patients with lung diseases facing increased ventilation pressure settings and low lung compliance, as well as the effect on ICP, since one patient developed an ICP increase directly after initiation of isoflurane.

Two previous prospective studies found no clinically relevant ICP increase up to 12 h after switching from intravenous to isoflurane sedation in a heterogeneous cohort of patients with acute brain injury (24, 25). In addition, a recent case series of seven patients with SAH who underwent decompressive craniectomy reported a slight/non-significant decrease of ICP in a 12 h follow up when patients received mono-isoflurane sedation due to insufficient sedation depth (26). So far, no severe adverse events after isoflurane administration in SAH patients have been reported; however, no study investigated long-term effects of volatile sedation with isoflurane in SAH patients beyond 12 h after isoflurane initiation.

In our series isoflurane was discontinued due to ICP increases (> 5 min) in five cases. While these ICP increases occurred mostly in the later course of treatment after a median of 30 h of ACD implementation, one patient developed an ICP elevation directly, within 10 min, after isoflurane was started. Noteworthy, episodic intracranial hypertension is a quite common phenomenon in the course of severe SAH as it is estimated that 50% of the patients experience ICP increases above 20 mmHg in acute (24 h), subacute (up to 7–10 days) and delayed stages after hemorrhage (33, 34). Therefore, the 11.1% of patients with documented episodes of increased ICP are in line with these findings. However, due to the lack of a control group, we cannot exclude a direct causal effect of low-dose isoflurane administration on ICP. Therefore, the use of balanced volatile sedation should be mitigated to SAH patients with an inadequate level of sedation using standard sedatives, and exclude patients with previous episodes of intracranial hypertension.

In contrast to our study, Purrucker et al. demonstrated a significant increase in ICP in the first hour after a complete switch from intravenous narcotics to sevoflurane in 25 patients with acute brain injury (21). In their study, ICP crisis that occurred in five patients was potentially attributed to a significant decrease in MAP and CPP caused by the vasodilatory effects of sevoflurane or an increase in *P*aCO_2_. Of note, in their study, two patients who exhibited ICP increases were switched from volatile sedation with sevoflurane to isoflurane instead and remained stable regarding their ICP thereafter.

In order to avoid hemodynamic instability, we focused on a balanced sedation regiment combining common intravenous sedatives with inhaled isoflurane at a lower concentration (MAC 0.18–0.5 for a 40-year-old standard patient) compared with the previous studies that targeted a MAC of ≥0.5. As expected, the addition of isoflurane sedation with intravenous hypnotic agents resulted in more adequate sedation depth measured with bispectral index. BIS correlates with common sedation scales such as RAAS and has already been reported to be useful for the differentiation of adequate from inadequate sedation depth in critically ill patients amongst others with SAH (35–38). As an additional advantage propofol dosage, which often is deemed indispensable in intravenous sedation protocols, could be reduced or even replaced by addition of isoflurane, thereby diminishing the imminent risk of propofol infusion syndrome (PRIS) (39).

Although our data also revealed a decrease in MAP and CPP in the first hour after initiation of isoflurane, none of the patients in our study dropped below the critical threshold of a CPP < 60 mmHg. After this short-term adaptation in blood pressure via increased norepinephrine dosage, the cardiovascular parameters remained stable in all patients until the end of isoflurane administration. These findings might favor a balanced sedation protocol over a complete switch to sevoflurane sedation in terms of hemodynamic stability, even though isoflurane is known for its more pronounced vasodilatory effect (40, 41).

Regarding the potential adverse effects of long-term isoflurane administration on extracerebral organ function, patients showed an increase in liver serum parameters, markers of muscle cell damage, and serum urea in long-term analysis. In one patient with a severe increase in serum liver enzyme concentrations, sedation with isoflurane was discontinued. However, other reasons for elevated serum parameters including liver enzymes, myoglobin and creatine kinase are possible and common, such as the SAH itself, toxicity by other drugs, systemic infections or the administration of parenteral nutrition (42–44). As isoflurane is a stable molecule only 0.2% is metabolized by the liver, and thus far, no clinical human studies have demonstrated hepatotoxicity or nephrotoxicity of low-dose volatile sedation in ICUs (43, 45, 46). Nevertheless, we cannot exclude a direct effect of volatile sedation with isoflurane on liver function in our cohort. Hence, patients with preexisting liver disease must be monitored with caution, when balanced sedation with isoflurane is in place. However, it needs to be considered that intravenous sedatives often exhibit detrimental liver toxicity, especially when administered in the inevitable high doses used in our cohort in order to reach sufficient sedation depth.

As already evidenced in previous studies, we observed a significant increase in *P*aCO_2_ on the first day after the implementation of the ACD. This might be attributed to an increased dead space in the ventilator system or the result of a known isoflurane-dependent increase in intrapulmonary shunt (47, 48). In the majority of patients, this increase in *P*aCO*_2_* could be counteracted by a significant increase in respiratory minute volume. However, isoflurane was discontinued in four patients due to uncontrollable hypercapnia. Data from our baseline comparison between patients with and without premature termination of isoflurane revealed that a pre-existing high peak inspiratory pressure > 20 cmH_2_O and a low lung compliance might serve as a predictor of pulmonary adverse events during isoflurane treatment. Although recent developments show a trend towards volatile vaporizers with lower dead space, volatile sedation in patients with SAH should be limited to patients without lung diseases and low ventilation pressure settings (49).

While providing new data specifically describing possible advantages of a balanced anesthesia protocol over mono-inhalational sedation, our study also has several limitations. First of all, it needs to be considered that establishing this balanced sedation protocol may not be feasible for all NICUs depending on the experience of the treating physicians with volatile anesthetic agents. Due to its retrospective nature, no confirmatory analysis was performed, and all *value of p*s are descriptive only. We did not include secondary outcome parameters other than death on NICU, since their interpretation would be misleading for several reasons: First, SAH patients in our NICU were often transferred into specialized neuro-rehabilitation and weaning centers for continuation of their intensive care treatment. Secondly, only relatively stable patients were considered to profit from balanced isoflurane sedation causing important selection bias. Isoflurane treatment was initiated after the individual decision of the treating physician; therefore, it was administered at different times after the onset of the hemorrhage. As SAH pathomechanisms vary over time, the current setup does not allow for speculation as to which component of SAH pathology is primarily influenced by isoflurane. Since there is no control group without isoflurane administration and due to the limited sample size, it is impossible to investigate whether outcome measures may be influenced by isoflurane treatment or to draw a conclusion regarding the safety of isoflurane treatment in these patients; however, our data help to better characterize the feasibility of long-term isoflurane sedation in patients with SAH. Hence, prospective studies investigating the safety of balanced volatile sedation in critically ill SAH patients are needed. Moreover, potentially favorable side effects on SAH in terms of putative neuroprotective effects versus its potential hazardous side effects can be elucidated.

In summary, a balanced sedation protocol including low-dose inhalational isoflurane via the Sedaconda^®^ ACD can achieve adequate sedation levels in some critically ill SAH patients. However, therapy had to be withdrawn in a substantial amount of patients mostly due to hypercapnia or ICP increase. Especially in patients with pre-existing lung injury, hemodynamic instability, and previous intracranial hypertension the balanced sedation protocol bears the risk of these adverse events. Therefore, the question if it is safe to use isoflurane in critically ill SAH patients remains unsolved and must consequently be addressed in a randomized trial.

## Data availability statement

The raw data supporting the conclusions of this article will be made available by the authors, without undue reservation.

## Ethics statement

The studies involving human participants were reviewed and approved by Ethical Committee of the Faculty of Medicine, LMU Munich, Pettenkoferstrasse 8a, 80336 Munich, Germany. Written informed consent for participation was not required for this study in accordance with the national legislation and the institutional requirements.

## Author contributions

MBM designed the study, analyzed and interpreted data and wrote the manuscript. NAT, SMS interpreted data, provided resources and revised the manuscript. JB critically edited the manuscript. VH designed the study, interpreted data, wrote the manuscript and supervised the study. All authors contributed to the article and approved the submitted version.

## Funding

Funded by the Deutsche Forschungsgemeinschaft (DFG, German Research Foundation)—413635475—and the Munich Clinician Scientist Program (MCSP) of the LMU Munich (MM).

## Conflict of interest

The authors declare that the research was conducted in the absence of any commercial or financial relationships that could be construed as a potential conflict of interest.

## Publisher’s note

All claims expressed in this article are solely those of the authors and do not necessarily represent those of their affiliated organizations, or those of the publisher, the editors and the reviewers. Any product that may be evaluated in this article, or claim that may be made by its manufacturer, is not guaranteed or endorsed by the publisher.
