# Supplementary material for: Balanced volatile sedation with isoflurane in critically ill patients with aneurysmal subarachnoid hemorrhage – a retrospective observational study
Source: Front Neurol. 2023 Jun 22;14:1164860. doi: 10.3389/fneur.2023.1164860 (PMC10324570; doi:10.3389/fneur.2023.1164860)
Supplement: Supplementary file 1 [file Table_1.DOCX]

Supplementary Material

Balanced volatile sedation with isoflurane in critically ill patients with aneurysmal subarachnoid hemorrhage – a retrospective observational study

Martin B. Müller^1^, Nicole A. Terpolilli^2^, Susanne M. Schwarzmaier^1^, Josef Briegel^1^, Volker Huge^3*^

*** Correspondence:** Corresponding Author: PD Dr. Volker Huge, MD; VHuge@schoen-klinik.de

| **Supplementary**  **table 1:** | **Comparison of baseline parameters between patients who tolerated volatile sedation and patients with premature termination of volatile sedation due to intracranial hypertension.** | | | | |
| --- | --- | --- | --- | --- | --- |
|  | **Parameter** | **Continued volatile sedation *n* = 11-27** | **Discontinued volatile sedation due to intracranial hypertension *n* = 5** | **Difference** | ***P*-value** |
|  |  | **(-24h to -1h)** | **(-24h to -1h)** |  |  |
| **Baseline parameters** | Age (years) | 56.04 (11.00) | 55.20 (8.44) | -0.84 (5.21) | 0.930 |
|  | Sex (m/f) | 7/20 (26/74%) | 1/4 (20/80%) | - | - |
|  | Duration (hours) | 288 [120 to 408] | 30 [7.5 to 120] | -258 | <0.001 |
|  | Fisher Scale | 4 [3 to 4] | 4 [4 to 4] | 0 | 0.296 |
|  | WFNS Score | 4 [2 to 5] | 5 [4 to 5] | 1 | 0.245 |
|  | ICU LOS | 32.52 (10.78) | 45.40 (16.07) | 12.88 (5.66) | 0.084 |
| **Cerebral monitoring** | ICP (mmHg) | 9.42 (2.78) | 8.74 (2.84) | -0.68 (1.36) | 0.617 |
|  | CPP (mmHg) | 80.69 (5.85) | 84.53 (5.14) | 3.84 (3.16) | 0.237 |
|  | BIS (Index) | 41.01 (9.11) | 44.27 (25.14) | 3.25 (7.24) | 0.659 |
| **Cardiovascular system** | HF (beats/min) | 64.03 (12.48) | 67.75 (5.54) | 3.72 (7.50) | 0.300 |
|  | MAP (mmHg) | 90.74 (5.65) | 95.48 (6.72) | 4.373 (3.70) | 0.220 |
|  | Temp (°C) | 36.90 (0.49) | 36.00 (0.95) | -0.90 (0.36) | 0.026 |
| **Respiratory function** | SaO_2_ (%) | 97.83 (1.45) | 99.10 (1.31) | 1.27 (0.91) | 0.185 |
|  | FiO_2_ | 37.60 (6.92) | 44.90 (6.16) | 7.30 (3.68) | 0.057 |
|  | RMV (L/min) | 8.30 (1.5) | 8.86 (3.68) | 0.55 (0.96) | 0.570 |
|  | Compliance | 58.46 (11.99) | 48.33 (16.05) | -10.14 (6.20) | 0.113 |
|  | maxP (cmH_2_O) | 19.80 (3.76) | 22.02 (3.28) | 2.21 (1.81) | 0.231 |
|  | PEEP (cmH_2_O) | 8.02 (1.92) | 7.93 (1.92) | 0.09 (0.94) | 0.924 |
|  | Horowitz index | 284.17 (73.74) | 354.2 (45.05) | 69.99 (34.32) | 0.051 |
|  | paO_2_ (mmHg) | 103.66 (17.41) | 119.20 (13.27) | 15.52 (8.24) | 0.069 |
| **Acid-base homeostasis** | pH | 7.42 (0.03) | 7.45 (0.06) | 0.03 (0.018) | 0.141 |
|  | paCO_2_ (mmHg) | 37.66 (2.24) | 36.13 (4.43) | -1.54 (1.29) | 0.231 |
|  | HCO_3_^-^ (mmol/L) | 24.73 (1.82) | 25.60 (2.40) | 0.88 (0.93) | 0.353 |
|  | Lactate (mmol/L) | 1.40 (0.73) | 1.06 (0.60) | -0.35 (0.35) | 0.324 |

**Supplementary table 1.** **Comparison of baseline parameters between patients who tolerated volatile sedation and patients with premature termination of volatile sedation due to intracranial hypertension.** M, male; f, female; duration, duration of volatile sedation; SAH, subarachnoid hemorrhage; Fisher Grade; WFNS grade, World Federation of Neurological Surgeons; LOS, ICU length of stay; ICP, intracranial pressure; CPP, cerebral perfusion pressure; BIS, bispectral index; HF, heart frequency; MAP, mean arterial pressure; Temp, body core temperature; SaO2, peripheral oxygen saturation; FiO2, fraction of inspired oxygen; RMV, respiratory minute volume; maxP, peak inspiratory pressure; PEEP, post endexpiratory pressure, Horowitz index, *P*aO_2_/FiO_2_; PaO_2_, arterial partial pressure of oxygen; PaCO_2_, arterial partial pressure of carbon dioxide; pH, blood pH in blood gas analysis; HCO3-, standard bicarbonate; n, number of patients with available data. Data represent means (±SD) or median (IQR) of all values given in the period (-24h to -1h before start of isoflurane).
